# Oxidative Stress, Antioxidant Cofactor Micronutrients, and Cognitive Outcomes in Childhood Obesity: Mechanisms, Evidence, and Therapeutic Opportunities

**DOI:** 10.3390/ijms262412012

**Published:** 2025-12-13

**Authors:** Marina Darenskaya, Karen J. Cloete, Luybov Rychkova, Sergey Kolesnikov, Zhanna Prokhorova, Natalya Semenova, Natalya Yuzvak, Lyubov Kolesnikova

**Affiliations:** 1 Scientific Centre for Family Health and Human Reproduction Problems, 664003 Irkutsk, Russia; rychkova.nc@gmail.com (L.R.); sikolesnikov2012@gmail.com (S.K.); 79148772181@yandex.ru (Z.P.); natkor_84@mail.ru (N.S.); iuzvak.n@yandex.ru (N.Y.); kolesnikova20121@mail.ru (L.K.); 2 UNESCO-UNISA Africa Chair in Nanosciences & Nanotechnology Laboratories, College of Graduate Studies, University of South Africa, Muckleneuk Ridge, P.O. Box 392, Pretoria 0003, South Africa; kaboutercloete@gmail.com

**Keywords:** childhood obesity, oxidative stress, antioxidants, mitochondrial dysfunction, hippocampus, cognitive outcomes, vitamins, micronutrients, phytopharmaceuticals

## Abstract

Overweight and obesity are major public health concerns among children and adolescents worldwide. The most prevalent form is exogenous–constitutional obesity, which is driven by a sedentary lifestyle and an unhealthy diet in which caloric intake exceeds energy expenditure. Beyond their association with chronic disease, these factors are closely linked to deficits in cognitive development and executive functions essential for learning (including working memory, sustained attention, planning, behavioral self-regulation, and cognitive flexibility). Oxidative stress (OS), characterized by the accumulation of reactive oxygen species (ROS) in cells and extracellular fluids, is a significant potential mediator in childhood obesity and an important contributor to its comorbidities. The antioxidant defense system (AOD)’s activity largely depends on levels of trace element cofactors, which determine the body’s resistance to adverse environmental factors (the “maladaptation phenomenon”). OS and trace element deficiencies contribute to the development of morphological changes in the brain, thus serving as a critical connecting link between childhood obesity and cognitive impairment. Non-pharmacological interventions are the most accessible and effective approach for prevention and treatment. Bioactive compounds derived from food and natural plants, classified as antioxidants and phytopreparations, may represent a promising complementary approach. These compounds are most effective when used in combination with sustained lifestyle modifications in children. Research in this area can help define future directions for study and develop targeted intervention strategies in the pediatric population. The aim of this review is to examine the relationship between OS, antioxidant cofactor micronutrients, and cognitive outcomes in childhood obesity and to explore mechanisms, evidence, and therapeutic opportunities.

## 1. Introduction

Over the last 30 years, overweight and obesity have been recognized as major public health concerns in younger generations [1]. Furthermore, statistical data indicate a steady increase in the prevalence of these conditions [2]. These negative trends contribute to the early development of a wide range of chronic diseases and complicate the management of pre-existing conditions [3]. Childhood and adolescent obesity significantly affect a child’s health by disrupting the function of nearly all systems (including the cardiovascular, nervous, endocrine, reproductive, and immune systems) [1], significantly increasing the risk of dyslipidemia, arterial hypertension (AH), diabetes mellitus (DM), non-alcoholic fatty liver disease, obstructive sleep apnea, and psychosocial disorders, all of which substantially reduce the child’s quality of life [4].

Urbanization is believed to promote the primary behavioral and environmental conditions that contribute to the growing proportion of children with obesity [1]. The most prevalent form of obesity is the exogenous–constitutional type, driven by physical inactivity and an unhealthy diet in which caloric intake exceeds energy expenditure [5]. A sedentary lifestyle, in addition to being a well-known risk factor for many chronic diseases, is closely linked to deficits in cognitive development and executive functions essential for learning (such as working memory, sustained attention, planning, behavioral self-regulation, and cognitive flexibility) [6,7]. These changes manifest at the behavioral, emotional, and personality levels in children with obesity, leading to poor socio-psychological adaptation to their environment [5,8].

An important potential mediator in obesity and its associated complications is the increased activity of OS reactions [9,10,11,12]. OS is characterized by the intense generation of ROS and nitrogen species, halogens, and active carbonyl compounds in cells and extracellular fluids, collectively known as ROS [13]. The accumulation of ROS shifts the balance of the free radical homeostasis system towards a predominance of oxidative reactions and can promote the development of OS [14]. The crucial role of chronic OS in obesity’s pathogenesis has been noted in children and adolescents [15].

OS can have both positive and negative effects and is therefore classified as oxidative eustress and oxidative distress [16]. In oxidative eustress, the increase in ROS levels is controlled by AOD mechanisms and associated with increased demand for cellular signaling and the activation of defense mechanisms in response to bacterial and viral pathogens [17]. In contrast, oxidative distress leads to an uncontrolled increase in ROS levels, causing irreversible oxidative modification of macromolecules, some of which become toxic, mutagenic, and carcinogenic [16].

OS can also act as a potential mediator not only for obesity but also for other diseases, making it a key link in multiple comorbidities [18,19,20]. Numerous studies have shown that bioelement deficiency in the human diet is associated with reduced resilience to adverse environmental factors, a phenomenon known as “maladaptation,” which involves an insufficient supply of essential substances [21]. At the level of free radical homeostasis, maladaptation manifests as a decrease in the concentration and activity of AOD enzymes for which bioelements serve as essential cofactors [22]. There is already evidence that OS in obesity can cause pathological changes, such as disruption of the blood–brain barrier, suppression of neurogenesis in the hippocampus, and reduced microvascular density, all of which may contribute to cognitive decline (including executive function, attention, working memory, processing speed, and academic outcomes) in children [23,24,25].

Excess weight in childhood and associated metabolic disorders often persist into adulthood. Therefore, early intervention to correct overweight in children and adolescents is necessary. Therapeutic options for childhood obesity remain limited, particularly in terms of pharmacological interventions. Drug therapy in this context includes only a few agents, which have several adverse effects. Consequently, the most accessible and effective approach for the prevention and treatment of obesity in children and adolescents is non-pharmacological intervention aimed at modifying the patient’s lifestyle and their environment.

While the relationship between OS reactions, micronutrient deficiency, and cognitive impairments in children with obesity has been proposed as a mechanistic link, the extent and nature of this relationship remain insufficiently characterized. An analysis of potential strategies for correcting these disorders is therefore highly relevant.

The aim of this review is to examine the relationship between OS, antioxidant cofactor micronutrients, and cognitive outcomes in childhood obesity and explore mechanisms, evidence, and therapeutic opportunities. This work can help define directions for future research and guide the development of effective interventions in children.

## 2. Pathogenesis of Childhood Obesity

Overweight and obesity in children are defined using percentile charts or standard deviation scores of body mass index (BMI), which account for the child’s height, weight, sex, and age [2]. The value of BMI in children changes with age, with high values in the first year of life, decreases in early childhood (2–5 years), and a gradual increase during puberty. Overweight in children is defined as BMI values deviating from the median in the range of +1.0 to +2.0 standard deviations, while obesity is defined as a deviation of more than +2.0 standard deviations [26]. Childhood obesity varies based on its etiology and severity, the presence of complications, and comorbid conditions. The diagnostic workup for children and adults with obesity is largely similar. Obesity can be either metabolically active, meaning it is accompanied by an increased risk of cardiovascular and other comorbidities, or metabolically benign, i.e., it does not carry a high risk of adverse outcomes [27].

In prepubertal and pubertal children, risk factors for developing obesity include having parents with obesity, a family history of obesity across three to four generations, a complicated obstetric history, early introduction of complementary foods, and body length above the 75th percentile at one year of age [28]. Maternal overweight or obesity, early formula feeding, parental history of obesity-related diseases (AH, metabolic syndrome, type 2 DM), and living in single-parent families are highly predictive for the development of obesity. Factors with moderate predictive value include birth length, high body weight at one year of age, and physical inactivity [29].

Childhood obesity is considered a polygenic and multifactorial disease in which the polymorphism of each gene, combined with environmental influences, determines the risk of development and the severity of the disease [1]. To date, approximately 22 genes associated with obesity risk have been identified, including genes involved in the synthesis of proteins such as leptin, melanocortin, and pro-inflammatory cytokines [30]. Abnormal eating behavior plays a leading role, with overfeeding being one of the primary causes of childhood obesity [27].

Adipose tissue, which consists of adipocytes, is now classified as an endocrine organ because it actively produces factors with endocrine, paracrine, and autocrine properties [31]. Adipocytes secrete a range of adipokines and pro-inflammatory molecules [32]. These biologically active components regulate the transmission of information about nutritional status to insulin-sensitive tissues and organs. In addition to an increased population of macrophages, the adipose tissue of children with obesity is infiltrated by immune cells and inflammatory lesions, such as adipocyte degeneration and fibrosis [32].

Hormonal imbalances in obesity are accompanied by elevated levels of leptin, resistin, visfatin, adipsin, and retinol-binding protein 4, among others [33,34]. The altered cytokine profile in obesity is characterized by an increase in pro-inflammatory cytokines and a decrease in anti-inflammatory cytokines, rendering obesity a subclinical chronic inflammatory condition with localized inflammation in adipose tissue [32]. There is an observed increase in interleukin (IL)-6, IL-1, and tumor necrosis factor (TNF-α)—known mediators of the early inflammatory response—as well as IL-8, interferon-γ, IL-18, and IL-1 receptor antagonist. The increased production of pro-inflammatory cytokines promotes the amplification of OS reactions [15].

The role and significance of gut microbiota alterations in the development of obesity have also been well established. Studies show that adolescents with obesity have an imbalance in the colonic microbiota, characterized by low levels of Bifidobacterium and Lactobacillus species, the presence of diverse Escherichia coli associations, and a high frequency of conditional pathogens and their consortia [35]. A reduction in microbial diversity and phylotype representation in the gut microbiome has been observed in adolescents with obesity and appears to depend on the duration of breastfeeding [36]. A significant difference has been found between the gut microbiota composition of obese and non-obese individuals, as patients with obesity show a predominance of Dorea (phylum *Firmicutes*), Bacteroides and Parabacteroides (phylum *Bacteroidetes*), and Slackia and Collinsella (phylum *Actinobacteria*) [37,38].

Due to the complex and multifactorial nature of the disease, research on the pathogenetic mechanisms underlying the formation and development of childhood obesity is gaining increasing importance.

## 3. Cognitive Impairment in Childhood Obesity

Analyses of cognitive disorders typically assess the state of a key area of the cerebral cortex—the hippocampus—responsible for learning, memory, and spatial orientation [39]. This region interacts with subcortical areas and the prefrontal cortex and plays a key role in executive functions. Therefore, hippocampal dysfunction leads not only to memory impairments but also to deficits in executive performance.

### 3.1. Animal Studies

Experimental studies have demonstrated alterations in hippocampal function in obesity [40]. It has been shown that a 16-week high-fat diet leads to disruptions in the histostructure of the hippocampus in animals of both sexes [41]. The most characteristic consequences of this diet include neurodystrophic changes in various areas of the hippocampus in male and female rats. Cognitive impairment was proportionate to the rats’ weight. A reduction in cell proliferation and the number of neuroblasts/immature neurons in the dentate gyrus of male mice also has been demonstrated [40]. Furthermore, histological damage has been observed, including degenerative neurons, damaged mitochondria, and dilated endoplasmic reticulum cisternae [42].

In a model of obesity induced by a high-calorie, choline-deficient diet, an increase in pro-inflammatory factors was noted in the rat cerebral cortex, including IL-2, macrophage colony-stimulating factor, macrophage inflammatory protein (MIP), and regulated on activation, normal T-cell expressed and presumably secreted (RANTES), along with a variably pronounced decrease in immunoregulatory cytokines (IL-10, IL-17A, IL-12p40, IL-12p70, TNF-α, MIP-2, and MIP-3α) [43].

Hyperglycemia and hyperlipidemia in obesity contribute to the development of endoplasmic reticulum stress in the hippocampus and the disruption of protein expression, which together impair learning and memory in rats [44]. It has been demonstrated that chronic exposure to a high-calorie, palatable diet may contribute to neuroinflammation, impairment of hippocampus-dependent memory, and increased glutamate levels in the hippocampus of obese mice [45]. It has been reported that male—but not female—rats with diet-induced visceral obesity exhibited depressive-like behavior [46]. Obese animal models consistently show worse performance in learning and memory tasks compared to non-obese animals [47]. High levels of glucose and saturated fatty acids have been shown to cause neuroinflammation, microglial activation, mitochondrial dysfunction, neuronal loss, and impaired synaptic plasticity [39]. Immunohistochemical studies in rats fed a high-fat diet demonstrated the recovery of hippocampal neurogenesis upon switching to a standard diet [41]. Potential mechanisms underlying the link between cognitive disorders and obesity include neuroinflammation, impaired vascular reactivity, and the influence of diet and obesity on myelination and dopamine function [48].

### 3.2. Adult Human Studies

Numerous studies in humans indicate a strong association between cognitive impairments and obesity [23]. Morphological changes in the brain contribute to cognitive decline, and increased body weight is associated with alterations in brain volume. It has been noted that individuals with overweight and obesity have reduced cortical thickness and gray matter volume [49]. With increasing BMI values, reductions in gray matter volume originated in the left caudate nucleus, medial orbitofrontal cortex, and left insula and expanded to the right hippocampus and left lateral orbitofrontal cortex and then to the right parahippocampal gyrus, left precuneus, and left dorsolateral prefrontal cortex [49]. These findings suggest that changes in gray matter volume in individuals with obesity may originate from reward/motivation processing regions, subsequently progressing to inhibitory control/learning memory regions [23,49]. Furthermore, obesity can cause brain inflammation, excessive gliosis, and, ultimately, depletion of brain cortisol reserves, significantly impairing learning and memory [23]. A cohort investigation revealed diminished gray matter volume and thickness correlated with elevated leptin levels in areas crucial for appetite regulation, decision making, and cognitive control, including the anterior insula, orbitofrontal cortex, and anterior cingulate cortex [50]. These findings suggest a potential adverse impact of heightened leptin concentrations on brain health and eating habits [50]. Moreover, changes in gray matter in obesity are associated not with total body fat percentage but its central distribution [51]. Dietary saturated fats have been found to promote the development of dementia later in life, and high-fat dairy products are associated with cognitive decline and an increased risk of cognitive impairment [52].

Memory impairments are among the most frequent cognitive dysfunctions associated with obesity and most often linked to biochemical changes. Several authors have correlated impaired spatial memory with hyperleptinemia and leptin resistance [53]. The latter is explained by the fact that leptin mediates the ionotropic glutamate receptor, inducing long-term potentiation via calcium influx, which is crucial for memory and learning [54]. The sustained inflammatory state in obesity disrupts coordinated communication between the periphery and the brain, which has a crucial role in maintaining homeostasis through humoral, nutrient-mediated, immune, and nervous signaling pathways. The inflammatory changes induced by obesity specifically affect communication interfaces, including the blood–brain barrier, the glymphatic system, and meninges [55]. Impairments in memory consolidation are linked to disrupted synthesis of neuropeptide Y in the hypothalamus, caused by an imbalance of the neurotransmitters ghrelin and leptin, leading to obesity. Because the hippocampus and the hypothalamus are in close anatomical proximity, these disruptions lead to impaired memory consolidation in the hippocampus and are associated with obesity [56].

### 3.3. Childhood Studies

In children with obesity, a high BMI is negatively correlated with gray matter volume in the prefrontal lobe and matrix-reasoning ability at baseline and after two years of follow-up [56]. Significant group and time effects in gray matter volume have been observed in the prefrontal lobe, thalamus, right precentral gyrus, caudate nucleus, and parahippocampal gyrus/amygdala. Young adults with obesity showed abnormal network connectivity metrics across extensive brain regions associated with the default mode network, the central executive network, and the salience network [57]. Young adults with obesity and metabolic syndrome—or its components—exhibit structural alterations in brain regions responsible for reward, cognitive control, and other functions, as well as changes in white matter integrity and volume [48]. Furthermore, children in this cohort show hyperreactivity in brain regions responsible for food reward and hyporeactivity in regions responsible for cognitive control during food-related tasks, as well as altered brain responses to food taste [58].

Numerous studies have reported changes in cognitive abilities in children with obesity [5,6,7,8]. Childhood obesity is associated with chronic persistent inflammation due to a pool of tissue macrophages that can penetrate the blood–brain barrier and cause neuroinflammation [59]. Data on the relationship between M2-polarized cluster of differentiation (CD)14^+^CD163^+^ peripheral blood monocytes, obesity in children, and neuropsychological deficiencies confirm the role of peripheral visceral obesity and neuroinflammation [59]. Three British cohorts were examined to identify possible links between early-life excess weight gain and cognitive performance in midlife [8]. The authors concluded that the association between early-life excess weight gain and lower cognitive performance in midlife is likely due to persistent low cognitive performance from childhood [8]. Childhood obesity is associated with deficits in cognitive abilities related to attention and cognitive flexibility [60]. Obesity is linked to worse academic grades—particularly in mathematics, reading, and executive functions—whereas physical fitness is associated with better cognitive performance, academic achievement, and behavior [60]. An indirect association was identified between higher BMI in children and impairments in mathematical ability, mediated by the influence of BMI on the child’s impulse control [61]. Preschool children with overweight or obesity performed significantly worse in all cognitive tests (reaction and movement time) compared to their normal-weight peers [62]. Children with obesity exhibit impaired executive functions, particularly in inhibitory control, cognitive flexibility, and sustained attention [6].

Children with obesity are more frequently diagnosed with attention-deficit/hyperactivity disorder (ADHD), and their intelligence quotient (IQ) and working memory index are often below the norm [63]. A meta-analysis of 34 studies revealed a significant correlation between obesity and reduced IQ—particularly verbal IQ—most evident in school-aged children [64]. Another meta-analysis of 13 studies showed that adolescents with obesity have reduced cognitive functions, including inhibitory control, memory, and cognitive flexibility, while physical exercise positively influences these domains but does not significantly affect attention [65]. Multivariate analysis of variance results showed that students with obesity performed worse on tests for reaction speed and visuospatial working memory than students with normal weight [66].

Children with obesity are also at increased risk of depression, anxiety, and low self-esteem, and these psychological problems often persist into adulthood, affecting overall quality of life [67]. Stigmatization and discrimination related to obesity further exacerbate these issues, leading to social isolation and mental health challenges, particularly among girls [68]. Weight-based teasing has been found to be closely associated with poorer emotional well-being in adolescents and an increased risk of suicidal tendencies [69]. Childhood obesity often affects a child’s socio-emotional development, leading to difficulties in social interaction and academic performance [67]. Obesity is frequently associated with negative body image, social isolation, and low self-esteem—all of which, combined with cognitive impairments, can significantly worsen a student’s overall well-being [7].

It has been noted that comprehensive interventions combining nutrition education with mindful, creative, and environmentally oriented physical activity can significantly improve cognitive functions, subjective well-being, and nutrition knowledge among overweight and obese lower-secondary school students [7]. Furthermore, while individuals with overweight exhibit cognitive impairments, such as mild cognitive decline, visuospatial deficits, and difficulties in memory and concentration, these impairments are generally less pronounced and less diverse than those associated with obesity.

## 4. OS in Childhood Obesity

One of the pathological changes observed in obesity is an excessive accumulation of ROS and resultant OS [11]. Across childhood cohorts, higher BMI/adiposity is consistently associated with higher circulating OS biomarkers [9]. An increase in BMI is associated with an increase in body fat mass, which contributes to adipose tissue dysfunction and its biochemical and biomechanical impact on surrounding tissues, leading to adverse metabolic consequences [9]. Preclinical studies have shown the stimulatory effect of OS on preadipocyte proliferation and differentiation, as well as an increase in adipocyte size [70].

In a healthy organism, ROS are involved in the activation of hypothalamic neurons that regulate eating behavior. In obesity, the enhancement of oxidative processes elevates ROS production and stimulates the hunger center [9]. This leads to an expansion of white adipose tissue depots and increased appetite. Factors stimulating OS reactions in obesity include hyperglycemia, dyslipidemia, micronutrient deficiency, chronic inflammation, hyperleptinemia, elevated muscle activity, endothelial dysfunction, and impaired mitochondrial respiration, among others [10,13]. Obesity is accompanied by an increased level of free fatty acids (FFA) in the blood, which stimulate the production of the superoxide anion radical [15].

The activation of OS reactions is typically mediated by several biochemical pathways, including superoxide generation by nicotinamide adenine dinucleotide phosphate (NADPH) oxidases, oxidative phosphorylation, protein kinase C activation, and the polyol and hexosamine pathways [13]. In obesity, mitochondria in adipocytes are among the main sources of ROS formation. Hyperleptinemia, decreased antioxidant activity, and chronic inflammation further intensify OS reactions [10]. OS progression leads to the accumulation of cytotoxic compounds in the body, including endogenous aldehydes, which act as mediators of cellular damage and provoke characteristic metabolic shifts [11,71]. These toxic products include malondialdehyde (MDA), 4-hydroxynonenal, 8-hydroxy-2′-deoxyguanosine (8-OHdG), and other oxidized nucleic acid bases and protein carbonyl groups [72,73,74].

Stimulators of OS in obesity include coexisting insulin resistance and hyperglycemia [9]. An intracellular increase in glucose levels activates glycolysis and the tricarboxylic acid cycle, increasing the production of oxidized forms of dehydrogenase coenzymes (NAD^+^ and flavin adenine dinucleotide (FAD)). This impairs mitochondrial respiratory chain function and results in the overproduction of the superoxide anion radical [74]. The accumulation of advanced glycation end products (AGEs) stimulates ROS production through the activation of NADPH oxidases and Nuclear Factor-kappa B (NF-κB). Because NF-κB serves as a transcriptional factor for adhesion molecules (E-selectin, endothelin-1, intercellular adhesion molecule-1), pro-inflammatory cytokines (IL-6, TNF-α), inducible nitric oxide synthase (iNOS), and microribonucleic acids (microRNAs), its activation leads to enhanced adipogenesis, inflammation, and OS [9,75]. Products of glucose auto-oxidation also serve as additional sources of hydroxyl and superoxide radical production [76].

Adipose tissue itself, as an endocrine organ, plays a significant role in the development of free radical pathology in obesity. It secretes pro-inflammatory cytokines and the enzyme aromatase into the internal environment, both of which are involved in maintaining free radical homeostasis [77]. Cytokines stimulate ROS synthesis, while aromatase participates in the synthesis of estrogens from androgens. Consequently, the hormonal profile shifts towards hyperestrogenism, and estrogens act as pro-oxidants, increasing the oxidative load on the body [34].

An additional stimulus for OS development in obesity is compensatory increased muscle activity, accompanied by progressive oxygen consumption, hypoxanthine accumulation in plasma, and overproduction of the superoxide anion radical [78].

In most cases, obesity disrupts lipid homeostasis, characterized by dyslipidemia associated with increased levels of low-density lipoproteins (LDLs) and decreased levels of high-density lipoproteins (HDLs) [79]. Both lipoproteins are potential substrates for oxidation, but LDL particles are oxidized significantly more readily than HDL, contributing to the progression of free radical oxidation processes [15].

It has been reported that hyperleptinemia maintains OS in obesity by increasing the production of hydrogen peroxide and hydroxyl radicals. Leptin promotes the proliferation and activation of monocytes and macrophages, accompanied by increased production of ROS and pro-inflammatory cytokines [80]. At the same time, leptin reduces the activity of antioxidant enzymes, such as intracellular paraoxonase (PON)-1—an enzyme that protects HDL from oxidation—leading to elevated levels of prostaglandin F2α, MDA, and hydroperoxides in blood plasma and urine [74].

Coenzyme Q10 (CoQ10), a key component of the mitochondrial respiratory chain and an endogenous antioxidant, can inactivate ROS both directly and indirectly through the activation of another antioxidant, α-tocopherol [81]. Reduced plasma coenzyme Q10 levels have been reported in children with obesity [81].

OS in obesity can cause deoxyribonucleic acid (DNA) damage and impair DNA repair, leading to the accumulation of damaged nucleic acids in cells, inflammation, altered gene expression, and disrupted cellular metabolism [82]. The degree of cellular genetic damage under OS is assessed based on the accumulation of oxidation products, including 8-OHdG [83]. Elevated levels of 8-OHdG have been reported in multiple studies in children and adolescents with obesity [84]. This marker is significantly higher in obese patients compared to individuals with normal weight [83]. Positive correlations between BMI and 8-OHdG concentration have been noted [85]. Associations between DNA damage and genomic instability in obesity—as well as a positive correlation between childhood obesity status and serum 8-OHdG levels—have been confirmed [84]. There is evidence that 8-OHdG can influence chromosomal stability by altering telomere maintenance. Telomere length in children with obesity was found to be approximately 23% shorter than in healthy peers, and telomere shortening was also associated with an increase in nuclear anomalies, such as the formation of nuclear buds and bridges. These data highlight the importance of monitoring 8-OHdG levels for predicting chromosomal instability in children and adolescents with obesity [11].

It has been shown that DNA damage can provoke inflammatory processes in visceral adipose tissue, leading to impaired lipid metabolism and systemic insulin resistance and exacerbating the course of obesity [9]. The accumulation of advanced oxidation protein products (AOPPs) serves as an indicator of extensive protein damage under OS activation. Evidence indicates that childhood and adolescent obesity is accompanied by increased AOPP levels [11]. The concentration of AGEs in the plasma of children with obesity was reported to be lower than in children with normal weight [86]. Accumulation of AGEs worsens free radical pathology through the activation of multiple mechanisms, mainly triggered by receptors for AGE product binding, which promote free radical formation [87].

The AOD plays a critical role in protecting against the damaging effects of OS. Its components stabilize biological membranes, inactivate free radicals, and prevent the propagation of chain free radical oxidation processes of organic compounds, primarily unsaturated tissue lipids [70]. This function is performed by non-enzymatic antioxidants, such as reduced glutathione (GSH), ascorbic acid, ceruloplasmin, ferritin, carnosine, tocopherols, retinol, and carotenoids, as well as a wide range of antioxidant enzymes, including superoxide dismutase (SOD), quinone oxidoreductase 1, catalase (CAT), glutathione peroxidases (GPx; eight isoforms), peroxiredoxins (six isoforms), glutathione-S-transferases (GST), glutathione-reductase (GR), aldo-keto reductases, and heme oxygenases [88].

Our previous studies established that adolescents with obesity exhibit disturbances in the thiol–disulfide system, including a decreased level of GSH against a background of increased oxidized glutathione [10,89]. Numerous studies have revealed decreased GPx activity in obese patients, as well as a positive association between GPx levels and BMI [71]. There is evidence that genetic variability in GST is associated with a predisposition to obesity and metabolic disorders. Reduced enzymatic activity of GST due to genetic polymorphisms may play a significant role in the development of OS in obesity [90]. GSH deficiency and the accumulation of oxidized compounds can have a cumulative effect, which may be associated with both inadequate GSH levels and increased demand for it as a cofactor for glutathione-dependent enzymes.

Clinical studies have demonstrated that children with obesity have reduced activity of antioxidant enzymes (SOD, GPx), decreased total plasma antioxidant status, and a positive correlation between these OS markers and BMI [91,92]. The balance between oxidative processes and AOD activity determines the intensity of metabolism and the organism’s adaptive capacity. The breakdown of the organism’s AOD is characterized by the development of an lipid peroxidation (LPO) syndrome, which can lead to several adverse cellular consequences, including membrane damage, enzyme inactivation, mitotic suppression, and accumulation of inert polymerization products [88].

## 5. Microelements as Cofactors of AOD Enzymes

Trace elements are essential chemical elements that maintain the homeostasis of the internal environment and perform numerous functions; they are integral components of enzymes, vitamins, hormones, and pigments. Stability of the body’s trace element composition is a prerequisite for optimal physiological function, corresponding to a specific period of ontogenesis [93]. Trace elements’ role as cofactors in regulating OS reaction enzymes is particularly significant [93].

SOD is a key AOD enzyme, and decreased SOD activity has been detected in adolescents with obesity [94]. The human body contains several SOD isoforms that are distinguished by their subcellular localization and, consequently, by the type of transition metal cofactor in their active site. The most common dimeric, Cu,Zn-SOD, is localized in the cytoplasm. It contains copper as the active-site cofactor and zinc, which stabilizes the enzyme’s conformation [95]. The second type, tetrameric Mn-SOD, is localized in the mitochondria of eukaryotic cells. The largest isoform, extracellular Cu,Zn-SOD, circulates in the extracellular space; it differs from the cytoplasmic form by its tetrameric structure and correspondingly higher molecular weight [96].

Beyond their role as cofactors in SOD, copper and zinc can activate metallothioneins—proteins whose antioxidant functions involve scavenging ROS and activating SOD, thereby increasing total antioxidant activity. Presumably, SOD activation occurs due to the ability of metallothioneins to release trace elements required for the enzyme’s tertiary structure [95]. Furthermore, several authors have reported that zinc is an important regulator of LPO intensity by stimulating SOD activity; accordingly, zinc deficiency leads to enhanced damage of cell membranes by free radicals [96].

The glutathione system, centered on the tripeptide glutathione and glutathione-dependent enzymes, represents another crucial component of protection against OS [97]. This system includes a family of enzymes that catalyze the reduction of organic peroxides using glutathione as an electron donor—GPx. There are eight known GPx isoforms, five of which contain selenium in their active site in the form of the amino acid residue selenocysteine. Selenium-containing GPx enzymes possess potent antioxidant properties, primarily reducing hydrogen peroxide to water; consequently, these enzymes neutralize LPO products, utilize hydrogen peroxide, and reduce hydroperoxides of fatty acids, proteins, and nucleic acids [93]. As a result, selenium deficiency leads to decreased activity and reduced serum concentrations of GPx, which can serve as an indicator of the body’s selenium status [96].

A similar mechanism operates in the thioredoxin (Trx) system, which includes Trx reductase enzymes that also contain selenium in their active sites. Three isoforms of this enzyme family are known: cytosolic (TrxR1), mitochondrial (TrxR2), and testis-specific (TrxR3). This system accepts electrons from NADPH via Trx reductases and transfers them to the active site of Trx, which then reduces disulfide bonds in proteins (e.g., glutathione) or other substrates. According to recent findings, the Trx system not only protects against oxidative damage to biomolecules but also participates in redox-dependent cell signaling using hydrogen peroxide and nitric oxygen (NO) [98]. It has also been observed that an increase in Trx reductase levels in tissues and blood serum correlates with elevated ROS production, suggesting the active participation of this system in counteracting the development of free radical oxidation processes [99].

Iron-containing enzymes of the AOD include the CAT and peroxidases, which contain trivalent iron [96]. Both enzymes decompose hydrogen peroxide into oxygen and water; however, peroxidases typically function at low hydrogen peroxide concentrations, whereas CAT operates at higher concentrations. Furthermore, CAT can remove peroxynitrite—formed from the reaction between NO and the superoxide anion radical—and counteract its formation by oxidizing NO to nitrite. Increasing evidence also suggests that CAT is involved in regulating physiological processes where hydrogen peroxide acts as a secondary messenger, oxidizing signaling molecules. These processes include cell signaling, proliferation, differentiation, and apoptosis [100].

It is important to note that transition metals can also act as pro-oxidants, initiating free radical reactions. For instance, iron can trigger LPO, and excessive accumulation of LPO products may lead to programmed cell death via ferroptosis [101].

## 6. Deficiency of Micronutrients Associated with OS and Childhood Obesity

Numerous studies on bioelements in children and adolescents with obesity have reported characteristic changes in the metabolism of essential chemical elements involved in free radical homeostasis [12]. For instance, obesity in children and adolescents is accompanied by an imbalance of copper in the body, with a pronounced decrease in this bioelement [102]. However, some studies have found no difference in copper concentrations compared to controls [103]. Yet, when stratified by sex and age, a significant association has been identified between the highest quartile of blood copper concentration and obesity status in children and adolescents [104]. Furthermore, in regression models, obesity was considered a negative predictor of copper content in hair [105]. Given that copper is involved in the functioning of the cardiovascular system, its deficiency can be presumed to be a risk factor contributing to the subsequent development of cardiovascular diseases in pediatric and adolescent patients [106].

In most studies, zinc levels were significantly lower compared to controls and negatively correlated with overweight and obesity [102]. However, the mean serum zinc level in children with obesity may still fall within the normal reference range [107]. Zinc content in hair samples was also inversely proportional to obesity [105].

Several other studies have found no significant differences in the levels of this bioelement among children and adolescents with normal BMI, overweight, and obesity [103]. Various trace elements of essential metals, including zinc, are directly involved in the synthesis, storage, and activity of insulin, forming a specific phenotypic subtype of insulin resistance that may represent one of the mechanistic pathways in the pathogenesis of obesity [108]. This may explain why zinc supplementation in this group can lead to a reduction in BMI, serum cholesterol, and LDL levels and, consequently, a decrease in oxidative processes [108]. It has also been suggested that zinc levels in childhood and adolescence may influence markers of cardiovascular disease and obesity [109].

Studies on selenium levels in obese patients without sex stratification showed a positive association with cholesterol levels in children and adolescents [104]. At the same time, plasma or serum GPx activity was reduced compared with the control group [110]. Conversely, another study revealed a decrease in the bioelement’s concentration, which was associated primarily with pre- and post-pubertal males [111]. In this case, it can be assumed that females have higher levels of this bioelement. However, in adulthood, obese women also exhibited a decrease in plasma and erythrocyte selenium concentrations alongside an increase in urinary selenium excretion. In binary logistic regression analysis, erythrocyte selenium was an independent predictor of serum cytokine IL-8 concentration in obese women, reflecting its anti-inflammatory effect in this group [112]. It follows that factors such as patient sex, age, and the type of biological material analyzed can influence selenium levels in the body. Depending on these factors, selenium may be redistributed for the regulation of carbohydrate and lipid metabolism, participation in immune response mechanisms, adipocyte differentiation, or the functioning of the AOD [110].

Changes in iron metabolism in children and adolescents with obesity are characterized by decreased serum iron levels, a reduced transferrin saturation index, and elevated ferritin and hepcidin levels. Positive correlations are observed between ferritin and prohepcidin levels and negative correlations between blood iron levels and hepcidin [113,114,115]. Elevated ferritin concentrations may reflect the development of systemic inflammation associated with obesity, occurring independently of the phenotype (metabolically healthy or unhealthy obesity) [116,117]. Thus, inhibition of intestinal absorption due to increased hepcidin expression in adipose tissue may be a primary cause of obesity-related iron deficiency, suggesting a classic regulatory mechanism for iron metabolism [113]. However, other relationships between iron metabolism parameters may also occur in obesity. Despite significantly lower serum iron levels, total iron-binding capacity, and transferrin saturation percentage, hepcidin and ferritin levels did not differ between the obese and control groups [118]. Other studies have shown no difference in iron content among children aged 4 to 16 years with normal BMI, overweight, and obesity. The authors suggested that obesity in adolescents has little effect on iron status but may influence erythropoietic activity [103,119]. When assessing gender-specific features of bioelement status in adolescents with obesity, a decrease in iron content was observed only in girls [120]. Based on these findings, it can be concluded that, in children and adolescents with obesity, in addition to physiological interrelationships between iron metabolism parameters, pathogenetic associations linked to sex may also develop [75].

Unlike other bioelements involved in free radical homeostasis, manganese levels in children with obesity were significantly higher compared to controls, with the highest values observed in individuals aged 6 to 19 years [9]. In an earlier study, conversely, no significant differences were found between groups in serum manganese levels, possibly due to the lack of sex stratification among participants [93]. This is supported by the finding that, when subjects were separated by sex, boys exhibited insufficient levels of manganese, as well as copper and zinc, relative to normal values. In contrast, older school-aged girls had elevated levels of manganese and zinc, with no significant differences in copper levels [121]. Other authors have also suggested the influence of sex and pubertal status on the content of essential metals in obesity [93,121].

Taken together, however, the evidence linking individual micronutrient deficiencies with childhood obesity and systemic OS remains predominantly observational and therefore largely correlative. Most studies are cross-sectional and based on single time point measurements of circulating minerals and vitamins, which are vulnerable to acute dietary variations, inflammation-induced redistribution, and assay variability, while key confounders, such as socio-economic status, overall diet quality, sleep, and physical activity, are incompletely captured. Recent large school-based surveys in India and other low- and middle-income settings illustrate this problem clearly; mixtures of ≥2 mineral deficiencies (iron, zinc, iodine, magnesium, selenium) are associated with decrements in attention, working memory, and visuospatial performance in children and adolescents, but these deficits cluster tightly with anemia, stunting, and low household resources, making it difficult to disentangle specific micronutrient effects from broader deprivation [122]. Moreover, micronutrient patterns in obese children are not mirror images of those in undernourished cohorts. For example, mild hyperferritinemia and copper excess may coexist with functional iron and zinc deficiency in the context of low-grade inflammation and hepatic steatosis.

Mechanistic studies in cell and animal models demonstrate that zinc, selenium, manganese, and magnesium deficits impair antioxidant enzyme systems, perturb mitochondrial redox balance, and promote adipocyte hypertrophy and ectopic lipid accumulation. However, human pediatric data rarely incorporate mechanistic biomarkers (e.g., enzyme activities, redox couples, or mitochondrial functional readouts). Interpreting the current literature therefore requires caution, as associations between low micronutrient concentrations and obesity-related OS in children are biologically plausible and internally consistent with mechanistic data but they cannot, in their present form, be assumed to reflect unidirectional causal pathways as opposed to reciprocal reinforcement between poor diet, adiposity, inflammation, and altered micronutrient handling.

## 7. The Link Between OS Parameters, Mitochondrial Dysfunction, Deficiency of Micronutrient Cofactors for AOD, and Cognitive Disorders in Childhood Obesity

An analysis of over 200 different observations showed that the brain, due to its high energy consumption and high content of redox-active metals, is particularly vulnerable to OS [123]. This is especially true for brain cells—neurons—that intensively consume oxygen, exhibit high metabolic activity, and possess a limited AOD. The unique structure of the brain, particularly its high concentration of polyunsaturated fatty acids, makes it especially susceptible to oxidative damage of key cellular biomolecules, including proteins, lipids, and DNA [124]. Activation of the LPO process leads to the formation of reactive aldehydes that further enhance OS and neuronal injury [125].

The brain is particularly sensitive to oxidative damage because of its high metabolic activity, characterized by intense oxygen consumption, dominance of oxidative phosphorylation, lack of energy reserves, high concentration of lipids prone to peroxidation, and elevated iron levels [126]. Mitochondrial homeostasis plays a crucial role in maintaining the energy balance of neurons and axons, and bioenergetic deficits significantly contribute to cognitive decline. ROS synthesis occurs in mitochondria, and when mitochondrial function is impaired, ROS production and OS intensify, while altered mitochondrial distribution disrupts neuronal axons’ energy homeostasis [127].

### 7.1. Mechanistic Structure: Hyperlipidemia/Hyperglycemia, ROS, Mitochondrial Permeability Transition Pore (mPTP), and Mitochondrial Quality Control

At a mechanistic level, hypernutrition, hyperglycemia, and hyperlipidemia impose a sustained substrate overload on mitochondria in insulin-sensitive tissues. Excess glucose and fatty acids increase the delivery of reducing equivalents (NADH, FADH_2_) to the electron transport chain (ETC), driving a highly reduced state of complexes I and III and promoting electron leak to molecular oxygen with consequent overproduction of superoxide and downstream ROS [128]. Under these conditions, mitochondrial Ca^2+^ uptake is also enhanced, both because of elevated cytosolic Ca^2+^ and direct modulation of the mitochondrial calcium uniporter. The combination of ROS and Ca^2+^ overload dramatically lowers the threshold for opening of the mPTP, a high-conductance channel in the inner mitochondrial membrane whose sustained opening collapses the proton motive force, induces matrix swelling, and leads to outer membrane rupture with the release of cytochrome c and other pro-apoptotic factors [129].

The mPTP is now recognized as a central integrator of metabolic and OS. Cyclophilin D, an intramitochondrial peptidyl-prolyl isomerase, sensitizes the pore to Ca^2+^ and ROS, while adenine nucleotides, Mg^2+^, and a high inner-membrane potential are inhibitory [130]. In obesity and type 2 DM, chronic hyperglycemia and elevated FFA increase matrix phosphate, induce subtle depolarization, and enhance LPO, all of which synergistically facilitate mPTP opening [128]. At the same time, the mitochondrial quality control (MQC) network, comprising mitochondrial biogenesis (Peroxisome proliferator-activated receptor gamma coactivator 1-alpha (PGC-1α)—nuclear respiratory factor 1/2–mitochondrial transcription factor A axis), dynamics (fusion via mitofusin 2 1/2 and optic atrophy 1; fission via Dystrophin-related protein 1), and mitophagy (PTEN Induced Kinase-1 (PINK1)–Parkin and receptor-mediated pathways), becomes progressively dysregulated [131]. Recent syntheses emphasize that in metabolic disease, persistent nutrient overload and ROS lead to fragmented, depolarized mitochondria that accumulate due to impaired mitophagy, while biogenic responses are blunted, producing a population of organelles with low respiratory capacity and high ROS output [132]. This self-reinforcing loop-substrate oversupply—ROS and Ca^2+^ loading, mPTP opening, and MQC failure—represents a plausible mechanistic bridge between childhood obesity, systemic OS, and evolving cognitive and neuropsychiatric sequelae.

### 7.2. Tissue-Specific Mitochondrial Responses in Metabolic Stress

Importantly, the mitochondrial response to hyperlipidemia and hyperglycemia is tissue-specific rather than uniform. In white and brown adipose tissue, nutrient overload promotes mitochondrial fragmentation, downregulation of oxidative phosphorylation, and reduced thermogenic capacity, changes that favor further fat accumulation and systemic insulin resistance [128]. In skeletal muscle, excess lipid delivery drives incomplete β-oxidation, accumulation of acylcarnitines, and increased mitochondrial ROS, leading to impaired insulin-stimulated glucose uptake and reduced exercise capacity. In the liver, mitochondrial dysfunction is closely linked to non-alcoholic fatty liver disease and steatohepatitis, where altered MQC and excessive mitophagy contribute to hepatocyte injury and fibrogenesis [133]. The developing brain appears particularly vulnerable, as diet-induced obesity in juvenile rodents causes region-specific reductions in respiratory chain complex activity, increased mitochondrial H_2_O_2_ production in the hippocampus and prefrontal cortex, and disruption of mitophagy, changes that correlate with impaired spatial memory, reduced hippocampal neurogenesis, and neuroinflammation [39]. Human imaging studies in obese youth, together with metabolic profiling of post-mortem brain tissue, likewise point to altered mitochondrial bioenergetics as a component of obesity-related brain insulin resistance and cognitive dysfunction, although direct functional measurements in pediatric populations remain scarce [134]. These data support a model in which systemic metabolic overload is translated into organ-specific trajectories of mitochondrial injury and repair, with brain mitochondria occupying a particularly narrow safety margin during critical windows of neurodevelopment.

In neuronal cells, mitochondrial dysfunction aggravates neurodegeneration and cell death, as mitochondria influence synaptic transmission, calcium homeostasis, neuronal plasticity, and morphological stability [135]. Under these conditions, the brain cannot generate sufficient energy due to impaired glucose transport or decreased efficiency of mitochondrial respiration [136,137,138].

Inflammation in obesity can increase the activity of lactate dehydrogenase, C-reactive protein (CRP), and plasminogen activator inhibitor-1 (PAI-1), thereby enhancing lactate oxidation, LDL expression in neurons, and mitochondrial ROS synthesis, leading to neurodegeneration and memory impairment [139]. Elevated PAI-1 levels impair the maturation of brain-derived neurotrophic factor (BDNF) in neurons via the c-Jun N-terminal kinases signaling pathway, rendering the brain more susceptible to neurotoxicity and cognitive decline [23]. Insulin resistance in brain regions with a high density of insulin receptors significantly affects mitochondrial efficiency and function [137]. Long-term consumption of a high-fat diet may contribute to impaired mitochondrial function in the brain [140].

For instance, in a diet-induced-obesity mouse model, the brains of 16-week-old mice exhibited opening of the mPTP, loss of mitochondrial membrane potential, and apoptosis, while insulin supplementation corrected these dysfunctions [39]. Experimental data show that in obesity, levels of MDA-lysine (MDAL) increase selectively in the frontal cortex (by 34%), and positive correlations were observed between MDAL levels, LDL, and body weight [141]. Moreover, increased lipid modification was associated with elevated levels of mitochondrial complex I (subunits NADH:ubiquinone oxidoreductase core subunit 3 and 9) and complex II (flavoprotein) [141].

Sirtuin-3 (SIRT3) significantly improves mitochondrial protein function, and SIRT3 suppression is a key component of metabolic syndrome predisposing to obesity. In mice with metabolic syndrome and SIRT3 gene deletion (SIRT3^−^/^−^) fed a Western diet, impaired mitochondrial respiration in the brain, enhanced insulin resistance, neuroinflammation, and β-amyloid plaque deposition were observed, along with reduced enzyme activity in several metabolic pathways, including fatty acid oxidation and the tricarboxylic acid cycle [142]. Studies have shown that elevated levels of biomarkers such as CRP, iNOS, and TNF-α in the brain lead to increased susceptibility to neurodegeneration [143].

Ferulate supplementation improved the serum lipid profile and antioxidant status and increased the mRNA expression of SOD1, SOD2, SOD3, CAT, and GP_X_ in animals’ frontal cortex and hippocampus [143]. Therapy using vagus nerve stimulation enhanced brain insulin sensitivity, reduced ROS production, alleviated mitochondrial dysfunction and cellular apoptosis, and consequently improved cognitive performance [144]. Increased levels of ROS, thiobarbituric acid-reactive substances, and AGEs, as well as elevated activity of GPx and GR enzymes, alongside decreased GSH and CAT activity and reduced brain-to-body weight ratio, were observed in the cerebral cortex of rats fed a high-fat diet for three weeks [145].

Results from a study on mice fed an obesity-inducing diet for 12 weeks showed alterations in the post-translational status of cytoskeletal, mitochondrial, and metabolic proteins [146]. Some of these proteins, such as phosphoglucomutase-1 and monocarboxylate transporter 1, were also implicated in brain dysfunction. The hypothalamus is a crucial brain region in the development of obesity, and its dysfunction is strongly associated with cognitive disorders [147]. In a study of diet-induced obesity in female APPswe/PS1dE9 (Alzheimer’s disease model mice, more pronounced obesity, insulin resistance, and hypothalamic mitochondrial dysfunction were observed compared with non-transgenic (wild-type) controls. Treatment with two NO donors—sodium nitrite or L-citrulline—did not reduce fat mass but improved total energy expenditure, substrate oxidation, and insulin sensitivity. Notably, both NO donors restored mitochondrial respiration in the hypothalamus of the Alzheimer’s disease model mice [147]. A significant correlation was also found between neurocognitive scores (visual memory, digit-symbol coding, Stroop test, continuous performance test, and attention-switching test) and levels of MDA and protein carbonyl groups [148].

Micronutrients play a key role in brain development and function; however, children and adolescents often fail to obtain sufficient micronutrients due to poor dietary habits [149]. Micronutrients, including copper, zinc, iron, and manganese, are linked to brain health through specific biological mechanisms [150]. Copper is a component of several enzyme systems and involved in neurotransmitter production [151]. Zinc acts as an essential cofactor in the nervous system, while copper, manganese, and iron determine inflammatory, oxidative, and stress responses in neurons [152]. However, some studies suggest that excessive serum levels of copper and iron may be associated with cognitive decline [153]. Inverse relationships have been identified between blood levels of magnesium and zinc and the prevalence of behavioral problems in children aged 6–18 years [154].

Regression analysis has shown that nutrient deficiencies—particularly of iron and iodine—combined with omega-3 fatty acid and vitamin D insufficiency predict lower cognitive performance in school-aged children [155]. Iron intake, primarily from red meat, nuts, and dark green leafy vegetables, was negatively associated with cognitive function [150]. Childhood obesity and overweight were associated with iron deficiency, elevated levels of CRP and glycated hemoglobin, and early cognitive decline linked to both iron deficiency and inflammatory factors [156]. Although these findings highlight some biological links between obesity, overweight, and cognitive function, further research is required to identify additional biological mechanisms influencing this relationship in the pediatric population.

It has been noted that iron deficiency anemia (IDA) adversely affects infants’, children’s, and adolescents’ motor, cognitive, social–emotional, and neurophysiological development in the short and long term [157]. IDA can lead to alterations in hippocampal energy metabolism, striatal dopamine turnover, and reduced myelination, which are associated with intellectual impairment [157]. Children with obesity frequently develop IDA, exhibiting lower hemoglobin, iron, and transferrin saturation levels and higher ferritin and hepcidin concentrations compared with non-obese peers [158]. Low selenium levels may also be associated with reduced cognitive function in children with obesity [3].

An equally important aspect is the existence of a complex system of interaction and regulation between the central nervous system (the brain), the enteric nervous system, and the gut microbiota, collectively referred to as the “gut–brain–microbiota axis” [159]. In this context, OS reactions and micronutrient deficiencies become critical, as they influence the regulation of digestive functions, the maintenance of mental health, and the normal functioning of the immune system [3].

From a translational standpoint, a structured view of mitochondrial dysfunction clarifies how OS and micronutrient disturbances could converge into neurocognitive outcomes in obese children. Experimental models indicate that obesity-associated mitochondrial ROS and mPTP opening in the hippocampus and the prefrontal cortex impair synaptic plasticity, reduce long-term potentiation, and disrupt insulin and BDNF signaling, thereby affecting learning, memory, and emotional regulation. Intriguingly, several pharmacological and lifestyle interventions that restore mitochondrial function, such as mitochondrial-targeted antioxidants, caloric restriction, structured exercise, or compounds enhancing mitophagy and biogenesis, improve cognition in high-fat-diet animal models even in the absence of major weight loss, suggesting that mitochondrial integrity is not only a downstream casualty but also a modifiable determinant of brain function. Yet, in human pediatric research, mitochondrial endpoints are rarely incorporated alongside cognitive assessments and micronutrient profiling. This mismatch between a sophisticated mechanistic framework and relatively rudimentary clinical phenotyping currently limits our ability to interpret associations between childhood obesity, micronutrient status, and neurobehavioral outcomes. Future pediatric trials that simultaneously track mitochondrial function, OS markers, and domain-specific cognitive performance are therefore essential to move the field from association to causation.

## 8. Clinical Relevance, Supplementation Trials, Methodological Limitations, and Emerging Mitochondrial-Targeted Therapies

Given that obesity is accompanied by serious physical and psychological complications, it is crucial to initiate treatment for children and adolescents with overweight or obesity in a timely manner. This requires acknowledging the multifactorial nature of the disease and employing a comprehensive approach. Non-pharmacological therapy is the primary treatment for childhood obesity. To prevent obesity, the importance of breastfeeding is emphasized first and foremost [160]. The basis of non-pharmacological treatment in children with obesity includes recommendations for lifestyle modifications (sleep–wake cycles), proper nutrition (reducing carbohydrates and animal fats while increasing vegetables and fruits), and physical activity (daily, moderate exercise) [7]. The diagnosis and correction of psychological problems should also be an essential component of non-pharmacological treatment for children with obesity [160]. Neurobiological studies have shown that aerobic exercise promotes angiogenesis and neurogenesis in the hippocampus and improves functional connectivity within neural networks responsible for working memory, enhancing activation in frontal, parietal, occipital, and limbic systems and the cerebellum [161]. A balanced diet rich in essential nutrients helps reduce OS and neurogenic inflammation [162].

The next step involves considering pharmacotherapy for weight loss in children. Typically, this approach is reserved for patients with severe obesity who are older than 10 years, have comorbid conditions, and have not responded to dietary therapy and lifestyle changes [163]. Medications in this category are few, costly, and have a high frequency of side effects.

Bioactive compounds derived from food and natural plants, classified as antioxidant agents and phytopharmaceuticals, can be used for the prevention and treatment of both childhood obesity and its associated cognitive disorders. These compounds may play an adjunctive role in combating childhood obesity by attenuating OS and inflammation [164]. It should be emphasized that the greatest efficacy of these compounds is likely achieved not through their isolated use but in conjunction with sustained lifestyle modifications in the child.

### 8.1. Observational vs. Interventional Evidence for Micronutrients and Cognition in Children

The distinction between observational associations and interventional evidence is critical when considering micronutrient-based strategies to mitigate cognitive sequelae of childhood obesity. Observational cohorts in diverse settings have repeatedly found that multiple micronutrient deficiencies, particularly those involving iron, zinc, iodine, and several vitamins, cluster with poorer scores in attention, executive function, and academic performance in school-aged children [165]. However, systematic reviews and meta-analyses of randomized trials indicate that the cognitive benefits of supplementation are, on average, modest and highly context-dependent. A landmark meta-analysis of multiple micronutrient trials in children reported only small standardized mean differences in fluid intelligence and academic achievement, with substantial heterogeneity between studies; benefits were greatest in younger and more severely deficient populations [166]. More recent syntheses incorporating physical activity and multi-nutrient interventions likewise conclude that while robust effects on cardiorespiratory fitness and some behavioral outcomes are consistently observed, micronutrient supplementation alone rarely produces large improvements in global cognition [167]. These discrepancies underscore that the impressive mechanistic plausibility of micronutrient–OS pathways does not automatically translate into large, generalizable gains in cognitive performance when examined in randomized pediatric trials.

### 8.2. Recent (2023–2025) Vitamin and Mineral Supplementation Trials Relevant to Cognition

Recent studies between 2023 and 2025 refine this picture but do not overturn it. Small pediatric studies suggest potential vascular or metabolic benefits of vitamin C (ascorbic acid) or E, but evidence for cognitive endpoints is limited and low-certainty; routine supplementation cannot be recommended outside of individualized, diet-first care [168]. Vitamin C is a water-soluble vitamin that is not synthesized in the body and therefore must be obtained through the diet. Its functional role is diverse; it is necessary for the synthesis of collagen, other proteins, and molecules, it acts as an electron donor (antioxidant), neutralizing free radicals, it functions as a reducing agent (enhancing iron absorption, transport, and retention), and it participates in the immune system [169,170]. It is well established that a diet rich in vegetables, fruits, and whole foods reduces the risk of weight gain, and vitamin C intake is associated with regulation of body weight and fat mass [164,168]. Overall, children with overweight and obesity tend to have lower levels of vitamin C, potentially associated with insufficient intake, altered distribution between muscle and adipose tissue, and gut dysbiosis [171]. Although studies on the efficacy of vitamin C supplements are limited, available data suggest that they may reduce blood leptin levels, body weight, and fat mass [160]. In children with obesity, vitamin C supplementation improved vascular status to control levels by improving blood flow regulation and reducing blood pressure [164]. Adequate vitamin C intake has been associated with improvements in memory, creativity, and mathematical abilities in children with obesity [172].

Like vitamin C, vitamin E may play a significant role in leptin metabolism and is functionally linked to obesity parameters [160]. Vitamin E is a fat-soluble vitamin involved in many biological processes, including the reduction of peroxyl radicals, forming a tocopheroxyl radical, which is then reduced by vitamin C. Thus, vitamin E maintains the integrity of long-chain polyunsaturated fatty acids in cell membranes and regulates the bioactivity of its components and lipid-related signaling pathways [173]. Among the eight vitamin E isomers, α-tocopherol and γ-tocopherol are the most common, known for their antioxidant, anti-inflammatory, and gene-regulating properties. α-tocopherol can activate the peroxisome proliferator-activated receptors (PPAR)-γ pathway, a key regulator of the production of the adipose tissue hormone adiponectin, which enhances fatty-acid oxidation, suppresses glucose production, and increases insulin sensitivity [174]. This vitamin also lowers cholesterol, which may help prevent the development of obesity [175]. Higher intake of folate, vitamin D, vitamin E, magnesium, zinc, and copper has been found to correlate positively with higher cognitive development levels in children [176].

Vitamin A can exist in two forms: preformed (all-trans-retinol and its esters) and provitamin A (β-carotene). Among these, β-carotene, like other dietary carotenoids, acts as an antioxidant substrate. Vitamin A is a fat-soluble vitamin that significantly influences development and metabolism, supports the immune system, and is closely linked to obesity parameters [177]. Higher levels of all serum carotenoids (α-carotene, β-carotene, α-cryptoxanthin, β-cryptoxanthin, and combined lutein/zeaxanthin) were associated with lower BMI and prevalence of obesity in children and adolescents [178]. Vitamin A status, along with CRP concentration, was associated with overweight and obesity in children and adolescents, especially in boys [179]. Reduced levels of retinoic acid are linked to the severity of neurodegenerative disorders, particularly autism in children, and vitamin A supplementation appears to be a promising approach for alleviating symptoms [180].

Vitamin D is a fat-soluble vitamin that is synthesized in the skin upon exposure to ultraviolet B radiation and hydroxylated to 25-hydroxycholecalciferol [25(OH)D], the accepted biomarker of vitamin D status. Vitamin D plays a crucial role in calcium and phosphorus metabolism in the skeletal system and is necessary for immune function. Maintaining adequate levels is important throughout childhood [160]. However, supplementation may also be beneficial for children with obesity, including amelioration of associated cognitive impairments [181]. Reasons for low vitamin D concentrations include dietary inadequacies, a sedentary lifestyle with less sun exposure, sequestration of vitamin D in the larger volume of adipose tissue, and impaired release from the skin into the bloodstream [182]. Although observational studies suggest that children with obesity may require higher vitamin D intake, definitive dosage recommendations remain under discussion [160]. Omega-3 supplementation at doses of 2 g/day and vitamin D at doses of 2000 IU/day have been evaluated in obese pediatric populations, with reported improvements in lipid profiles, inflammatory markers, and insulin sensitivity. However, given the variability in study design, duration, and participant characteristics, these doses should not be generalized without clinical supervision [183]. Higher doses of vitamin D (1000–1500 IU/day) during autumn and winter are recommended for children with obesity or year-round in case of insufficient sun exposure [183].

Some researchers attribute the weight loss associated with vitamin D intake to its anti-inflammatory effects [181]. Post hoc analyses of vitamin D supplementation trials in pregnancy and infancy suggest that higher concurrent 25(OH)D levels in early childhood are associated with slightly better language or global developmental scores, yet randomization to higher versus standard-dose vitamin D_3_ (e.g., 1200 vs. 400 IU/d in the first two years of life or 2800 vs. 400 IU/d in late pregnancy) has not yielded consistent improvements in standardized neurodevelopmental test scores up to 6–8 years of age [184]. A post hoc analysis of one such cohort reported that contemporaneous 25(OH)D concentrations and maternal education predicted Brigance quotient scores more strongly than prenatal treatment assignment, highlighting the influence of social determinants and ongoing nutritional status [185]. The cognitive index of children with obesity is lower than that of children with normal weight, and lower levels of 25(OH)D are associated with cognitive deficits in children with obesity [186,187]. Children under 2 years old with lower serum vitamin D levels (average 27.65 ng/mL) have significantly lower scores in the problem-solving domain of the American Society for Quality-3 [188]. In contrast, targeted vitamin D_3_ supplementation (typically 50,000 IU/week for 8–12 weeks) in children with ADHD has been shown to modulate quantitative electroencephalography patterns and improve clinical symptom scores when combined with neurofeedback, suggesting that in selected neuropsychiatric phenotypes with low baseline vitamin D, physiological and electrophysiological benefits may be detectable over short timescales [189]. It has been established that levels of vitamin D, iron, omega-3 fatty acids, homocysteine, CRP, BDNF, glucose, insulin, and antioxidants can provide valuable information on the influence of nutrition on cognitive development [162,190,191].

Beyond vitamin D, multi-micronutrient formulations have been tested in several neurodevelopmental contexts. An 8-week, three-site, randomized controlled trial of broad-spectrum micronutrients in unmedicated children with ADHD showed moderate improvements in inattentive and emotional symptoms compared with a placebo, although effects on objective cognitive tests were smaller [192]. More recently, a quasi-experimental study combining a multi-micronutrient nutritional formula with aerobic exercise in children with ADHD reported gains in executive function (measured by Stroop and Wisconsin Card Sorting tests), creativity, and sleep quality, but the non-randomized design and potential practice effects on cognitive tasks preclude firm causal inference [193]. School-based cluster trials integrating multi-micronutrient supplementation into broader physical activity programs suggest that cognitive benefits are again most apparent in children who are frankly deficient at baseline, whereas in generally well-nourished cohorts the effect of supplementation on cognitive outcomes is small or absent [194]. Notably, almost none of these trials have focused specifically on obese children, and OS or mitochondrial biomarkers are rarely measured, limiting their direct translatability to childhood obesity-related cognitive risk.

B vitamins are also involved in regulating the AOD. During childhood and adolescence, adequate blood levels of these vitamins are necessary for nucleic acid synthesis, cell division, amino acid and lipid homeostasis, erythrocyte formation, and nervous system myelination [195]. Children with overweight, obesity, and metabolic syndrome have a greater risk of mineral and vitamin deficiencies [196]. A logistic regression model identified potential interactions between vitamin B2 and vitamin B12 that may increase the risk of obesity in children and adolescents [178]. These vitamins are primarily used in combination and are employed for treating childhood conditions such as migraine, pain syndromes, and ADHD. Specifically, vitamin B2 (riboflavin) is a cofactor in redox reactions within the electron transport chain, plays a key role in mitochondrial energy production, and may influence recovery from mitochondrial dysfunction associated with migraine [197]. Vitamin B6 regulates the synthesis and metabolism of 5-hydroxytryptamine, serotonin receptors, and catecholamines; it is widely used to treat behavioral disorders in children and can enhance the efficacy of antiepileptic drugs [194]. Numerous studies have focused on the effectiveness of vitamin B6 in combination with magnesium (Mg) for managing stress during adolescence; additionally, pyridoxine reduces peripheral corticosteroid release and influences the central biosynthesis of various neurotransmitters associated with depression and anxiety [198]. Vitamin B12 deficiency is more common in obese children than in healthy controls, regardless of metabolic phenotype [199].

Metal ions (including copper, zinc, selenium, iron, manganese, and others) are essential cofactors for important antioxidant enzymes and used as dietary supplements. Adequate intake is necessary for the inducers of these enzymes to reach effective activity levels. Copper and zinc play an important role in preventing superoxide damage through their involvement in Cu/Zn-SOD, which may help reduce the risk of obesity [200]. Furthermore, zinc acts as a key participant in metabolic and endocrine processes, immune function, and other reactions [160]. Zinc also plays a crucial role in the synthesis, storage, and release of insulin, and its deficiency can contribute to the development of insulin resistance, DM, atherosclerosis, and coronary heart disease [164]. Even a slight reduction in zinc levels may promote the development of obesity [104].

Selenium is a vital element and a cofactor for enzymes with antioxidant functions. It promotes the production of selenoprotein P1, which regulates the Kelch-like ECH-associated protein 1/Nuclear factor erythroid 2-related factor 2 pathway. Selenium supplementation has a positive effect on body weight regulation, metabolic functions, and OS, similarly to zinc [201]. The relationship between selenium levels and childhood obesity involves interactions with the AOD, thyroid hormone metabolism, and inflammation; therefore, maintaining adequate selenium levels may help mitigate some of the negative metabolic consequences of childhood obesity. It has been found that selenium (Se) supports healthy brain neurodevelopment and cognitive function by improving synaptic plasticity, regulating Zn^2+^ levels and autophagy, and inhibiting ferroptosis [202]. Preschool children with selenium deficiency scored lower on cognitive tests than typical children, and school-aged children demonstrated poorer cognitive outcomes, such as developmental delays and low academic achievement [123]. The balance between selenium intake, absorption, and metabolism is vital, reflecting the complex interrelationships between nutrition, OS, obesity, and cognitive impairment in children [3].

Children with obesity have demonstrated a higher frequency of anemia, low hemoglobin, and median serum iron [203]. A connection exists between this state, insulin resistance, and obesity, which may be driven by inflammation. Obesity in children and adolescents may also be associated with lower IQ, diminished cognitive abilities, and impairments in motor and visuospatial skills, where elevated levels of hepcidin—a key regulator of systemic iron homeostasis—may play a decisive role [164]. Iron supplementation has shown a significant positive effect on the intelligence, attention, concentration, and memory of school-aged children; however, data on the impact of iron supplements on academic performance are limited [204]. Iron status and anemia may be linked to academic performance in some cases, and iron supplementation during adolescence may improve academic achievement, attention, and concentration. However, nearly all studies on iron supplementation were judged to have a moderate to high risk of bias [205].

Magnesium (Mg) plays a critical role as a cofactor for enzymes responsible for carbohydrate metabolism and is particularly crucial for insulin action. Hypomagnesemia is also associated with obesity, although it remains unclear whether it is a cause or a consequence of metabolic dysfunction. In individuals with obesity, low intracellular magnesium levels can impair glucose oxidation and lead to excessive NADPH production, which promotes triglyceride synthesis and fat accumulation in adipocytes and exacerbates obesity. Research suggests that optimizing magnesium levels may enhance the positive metabolic effects of vitamin D and reduce the likelihood of obesity-related comorbidities. Thus, the relationship between magnesium and vitamin D in obesity underscores their combined influence on the pathogenesis and treatment of metabolic diseases [206]. Magnesium deficiency may contribute to the early development of insulin resistance in childhood obesity, warranting further investigation into the mechanisms underlying this phenomenon and potential therapeutic interventions [146]. Low dietary magnesium intake may play a role in the etiology of behavioral disorders in young children [206].

Phytopharmaceuticals include dietary antioxidants, such as resveratrol, quercetin, coenzyme Q10 (CoQ10), curcumin, astaxanthin, and other compounds. They are abundantly found in berries, vegetables, fruits, grapes, turmeric, and seafood and exert complex systemic effects [70]. These substances can improve mitochondrial dysfunction to alleviate metabolic disorders [207]. Targeting mitochondrial dysfunction and OS may be a potential mediator in treating obesity-related cognitive dysfunction. A network meta-analysis of 48 studies involving 12 antioxidant agents (resveratrol, pycnogenol, omega-3, omega-6, quercetin, phosphatidylserine, almonds, vitamin D, zinc, folic acid, ginkgo biloba, and acetyl-L-carnitine) with 3650 participants noted the safety of omega-6, vitamin D, and quercetin [208]. Optimal agents for improving attention, reducing hyperactivity, and improving total scores on the Parent-Rated ADHD scale were phosphatidylserine, resveratrol + methylpyrrolidine, and phosphatidylserine, respectively. Optimal agents for improving attention, hyperactivity, and total scores on the Teacher-Rated ADHD scale were pycnogenol, vitamin D, and vitamin D, respectively. Resveratrol has been shown to protect dopaminergic neurons from OS-induced apoptosis [160]. Natural compounds like quercetin and astaxanthin possess antioxidant properties and can reduce oxidative damage in the brain. Quercetin enhances the brain’s ability to mitigate OS-induced toxicity by modulating PON-2 levels [209]. Astaxanthin has been shown to slow brain aging by reducing OS and increasing levels of BDNF, which is essential for neuronal health and function [210].

Omega-3 polyunsaturated fatty acids have garnered attention as a potential adjunctive treatment for ADHD due to their role in cognitive and neurobiological functions, such as attention and concentration, impulse control, executive functions, working memory, neurotransmitter regulation, and brain development, primarily of the prefrontal cortex [211]. Specifically, eicosapentaenoic acid and docosahexaenoic acid have demonstrated efficacy in alleviating cognitive disorders and improving metabolic health [212]. By modulating gut microbiota, reducing inflammation, and influencing neurotransmitter systems, omega-3 polyunsaturated fatty acid may offer a novel approach to treating these disorders in children [213].

Co Q10 (ubiquinol, ubiquinone) is often referred to as a vitamin-like substance, although it is not a vitamin as it is produced by various tissues in the human organism [81]. CoQ10 is an essential component of the mitochondrial electron transport chain involved in Adenosine triphosphate production via oxidative phosphorylation in mitochondria. It is a crucial antioxidant that acts as a cofactor for mitochondrial uncoupling proteins and inhibiting LPO, prevents LDL oxidation, enhances the bioavailability of other antioxidants, including vitamins C, E, and β-carotene, participates in the metabolism of fatty acids, amino acids, and cholesterol, and directly influences the expression of numerous genes, including those involved in inflammatory processes [81].

N-acetylcysteine (NAC) is considered an effective antioxidant and anti-inflammatory agent that exhibits neuroprotective properties through the restoration of glutathione [123]. Specifically, NAC has demonstrated improvements in bioenergetics and behavioral responses following traumatic brain injury [123]. NAC supplementation has been noted to protect against high-fat-diet-induced weight gain, hyperglycemia, and insulin resistance. Furthermore, NAC improved redox and inflammatory status in the cerebral cortex [214]. In an experimental model of obesity, the combination of NAC and taurine prevented hippocampal alterations and memory impairment [215]. NAC, which stimulates glutathione biosynthesis, may attenuate ferroptosis in the hippocampus in obesity by suppressing the downregulation of GPX4 and solute carrier family 7 member 11 expression [216]. Although further research is needed to investigate these effects in humans, these studies suggest that treatments like NAC supplementation, which reduces OS and improves mitochondrial function, could be a simple and effective way to support brain health [39].

Melatonin, a neurohormone traditionally known for regulating circadian rhythms and sleep, also possesses potent antioxidant, anti-inflammatory, and neuroprotective properties [217]. Preclinical studies indicate that melatonin reduces OS and inflammation and improves endothelial function and metabolic dysfunction, including insulin resistance and impaired lipid metabolism [218,219]. Melatonin has demonstrated potential in preventing cognitive decline by reducing oxidative damage in the hippocampus, preserving synaptic plasticity, and enhancing neurogenesis [220]. These neuroprotective effects may counteract the cognitive impairments often observed in patients with obstructive sleep apnea [220]. Melatonin supplementation (1–20 mg per day) has been shown to reduce mitochondrial damage, improve glucose regulation, and increase brown adipose tissue activity in childhood obesity without significant side effects. Furthermore, the antioxidant properties of melatonin have been shown to reduce exercise-induced muscle damage in individuals with overweight [156].

Astaxanthin, a carotenoid biomolecule derived from natural sources (e.g., microalgae), exerts a range of health benefits due to its strong antioxidant and anti-inflammatory capacities, which can influence various brain processes and alleviate symptoms [221]. Astaxanthin intake significantly reduces the accumulation of ROS, limits neuronal apoptosis, and mitigates neurodevelopmental impairments [222]. Astaxanthin activates the activated protein kinase signaling pathway and serves as a typical PPARα agonist, enabling it to regulate energy metabolism and promote lipid metabolism in the body. It enhances insulin sensitivity and accelerates glucose metabolism by activating the Phosphatidylinositol-3-kinase/Protein kinase B signaling pathway, thereby alleviating symptoms of obesity, hyperglycemia, and dyslipidemia, and exerts beneficial effects on gut flora and its metabolites [223].

### 8.3. Critical Appraisal of Supplementation Trials

Several methodological features help explain why the clinical effects of vitamin and mineral supplementation on cognition appear weaker and more inconsistent than suggested by mechanistic and observational work. First, many pediatric trials recruit broadly healthy children with relatively low prevalence of severe deficiency; under these circumstances, both low- and high-dose regimens may succeed in maintaining 25(OH)D or trace mineral concentrations within an adequate range, resulting in minimal between-group biological differences [184]. Second, dosage and duration vary widely, ranging from high-dose “loading” protocols over 8–12 weeks to low-dose maintenance regimens over several years, yet few studies systematically explore dose–response relationships or stratify by baseline status. Recent structured reviews of the vitamin D–BDNF–cognition axis in adults emphasize that doses ≥ 2000 IU/d or 50,000 IU/week targeted at frankly deficient individuals are most likely to influence neurotrophic markers and mood, whereas evidence for cognitive benefits at lower doses or in replete populations is sparse [187]. Whether analogous thresholds exist in children, particularly in the context of obesity-related sequestration of fat-soluble vitamins, remains unknown. Third, cognitive endpoints themselves are heterogeneous, ranging from screening tools (e.g., Ages and Stages Questionnaire3, Brigance) to full-scale IQ batteries and domain-specific executive function tests, often administered at ages when test–retest reliability and motivational factors (fatigue, subclinical mood symptoms) can strongly influence performance [187]. Finally, few trials incorporate long-term follow-up into adolescence, when obesity-related structural and functional brain changes become more pronounced and might be more sensitive to sustained improvements in micronutrient status and OS.

Age-related developmental windows also require explicit consideration. Nutritional meta-analyses and long-term follow-ups of preconception and prenatal multiple-micronutrient supplementation trials suggest that the largest and most durable cognitive gains are achieved when deficits are corrected before or during pregnancy and in the first thousand days of life, whereas supplementation initiated later in childhood tends to produce smaller effects. For childhood obesity, this implies that interventions aimed at normalizing micronutrient status and mitigating OS may have the greatest neurodevelopmental impact when implemented early, ideally before the combination of adiposity, systemic inflammation, and mitochondrial dysfunction becomes entrenched.

### 8.4. Emerging Mitochondrial-Targeted Strategies

Beyond classic vitamin and mineral supplementation, several emerging therapeutic approaches directly target mitochondrial function and may, in principle, be relevant to obesity-related cognitive impairment. Inhibition of the mPTP is one such strategy. Cyclosporin A and non-immunosuppressive cyclosporin analogs (e.g., NIM811, sanglifehrin A) bind to cyclophilin D and raise the threshold for Ca^2+^- and ROS-induced pore opening, thereby preserving mitochondrial membrane potential and limiting cell death in models of ischemia–reperfusion injury and neurodegeneration [224]. While these agents have mainly been explored in adult cardiovascular and neuromuscular disease and no childhood obesity trials exist, they provide proof of concept that pharmacological modulation of mPTP-dependent pathways is feasible in humans. Parallel efforts focus on mitochondrial ion transport; opening large conductance mitochondrial K^+^ channels and modulation of Ca^2+^ influx via the mitochondrial calcium uniporter can reduce ROS production and limit mPTP opening in preclinical models, though specificity and safety remain major obstacles to translation.

Another promising avenue is the use of mitochondria-targeted antioxidants and mitophagy/biogenesis modulators. Compounds such as elamipretide, a cardiolipin-binding peptide recently approved for Barth syndrome, improve ETC efficiency and reduce mitochondrial ROS in patients with primary mitochondrial disease and in animal models of metabolic stress [225]. Small molecules that enhance mitophagy (e.g., urolithin A) or activate PGC-1α-dependent mitochondrial biogenesis (e.g., adenosine monophosphate-activated protein kinase activators, some polyphenols) have shown the capacity to normalize mitochondrial morphology, reduce oxidative damage, and improve cognition in rodent models of high-fat-diet-induced obesity and neurodegeneration [226]. Importantly, these interventions do not primarily act by reducing body weight; their impacts on synaptic function and behavior often precede or exceed changes in adiposity, strengthening the argument that mitochondria are actionable nodes in the causal chain linking obesity to brain dysfunction. At present, however, virtually all such data are derived from adult or preclinical studies, and pediatric safety, optimal dosing, and long-term developmental effects are unknown. Given these uncertainties, mitochondrial-targeted agents should currently be viewed not as imminent clinical tools for obese children but as mechanistically informative probes that can help dissect the contribution of mitochondrial pathways in carefully designed translational studies.

### 8.5. Methodological Considerations and Sources of Heterogeneity

The inconsistencies across studies summarized in Section 6, Section 7 and Section 8 can be traced to recurring methodological issues that merit explicit acknowledgment. Most human data linking micronutrients, OS, and cognition in children are derived from observational cohorts in which micronutrient exposures are assessed once or twice, while cognitive outcomes are influenced by a dense web of correlated factors, including socio-economic status, parental education, home cognitive stimulation, sleep, and co-morbid conditions, such as ADHD, epilepsy, and mood disorders [122]. Even when sophisticated multivariable models are used, residual confounding and reverse causality (e.g., obesity and inflammation lowering circulating micronutrient levels) remain plausible explanations for observed associations. In contrast, randomized supplementation trials typically offer stronger internal validity but at the cost of narrower inclusion criteria, shorter follow-up, and often inadequate biological contrasts between intervention arms. Meta-analyses that pool such heterogeneous designs inevitably show substantial between-study variance, and summarized effect sizes, while statistically informative, can conceal subgroups in whom micronutrient interventions are either particularly effective (severely deficient, high OS burden) or essentially redundant. Across both observational and interventional work, the near absence of mechanistic biomarkers (e.g., mitochondrial function, detailed redox profiling, or neuroimaging markers of brain metabolism) limits the capacity to test whether improvements in micronutrient status actually modify the oxidative and mitochondrial pathways hypothesized to drive cognitive risk in childhood obesity.

## 9. Conclusions

In summary, the available literature strongly supports a mechanistic framework in which nutrient excess, micronutrient imbalance, and OS converge on mitochondrial dysfunction—particularly mPTP dysregulation and impaired mitochondrial quality control—to threaten neurodevelopment in children with obesity. However, the human evidence base is characterized by sharp asymmetry, including rich mechanistic and animal data, numerous observational associations, and comparatively few methodologically rigorous pediatric trials that integrate micronutrient interventions with mitochondrial and cognitive endpoints. Recent randomized studies of vitamin D and multi-micronutrient supplementation, including those published between 2023 and 2025, suggest that clinically meaningful cognitive benefits are most likely in settings of frank deficiency, high oxidative burden, and early developmental windows, whereas effects in generally replete populations are small and inconsistent [184].

Future work should therefore prioritize (i) longitudinal cohorts of obese and overweight children with repeated assessments of micronutrients, oxidative and mitochondrial biomarkers, and domain-specific cognitive outcomes; (ii) randomized trials that target clearly defined deficiency states, use adequately powered contrasts in dose and duration, and incorporate mechanistic readouts, such as mitochondrial function and redox status; and (iii) careful exploration, in age-appropriate models, of emerging mitochondrial-directed therapies as adjuncts rather than alternatives to lifestyle and dietary interventions. Only by explicitly integrating observational, mechanistic, and interventional evidence in this way can the field move from associative claims towards a robust, causally grounded understanding of how micronutrient status and OS shape cognitive trajectories in children living with obesity.

## Data Availability

No new data were created or analyzed in this study. Data sharing is not applicable to this article.

## Abbreviations

The following abbreviations are used in this manuscript:

OS	Oxidative stress
ROS	Reactive oxygen species
AOD	Antioxidant defense system
AH	Arterial hypertension
DM	Diabetes mellitus
BMI	Body mass index
IL	Interleukin
TNF-α	Tumor necrosis factor-alpha
MIP	Macrophage inflammatory protein
RANTES	Regulated on activation, normal T-cell expressed and presumably secreted
CD	Cluster of differentiation
ADHD	Attention-deficit/hyperactivity disorder
IQ	Intelligence quotient
FFA	Free fatty acids
NADPH	Nicotinamide adenine dinucleotide phosphate
MDA	Malondialdehyde
8-OHdG	8-hydroxy-2′-deoxyguanosine
NAD	Nicotinamide adenine dinucleotide
AGEs	Advanced glycation end products
NF-κB	Nuclear Factor-kappa B
iNOS	Inducible nitric oxide synthase
LDL	Low-density lipoproteins
HDL	High-density lipoproteins
PON	Paraoxonase
CoQ10	Coenzyme Q10
DNA	Deoxyribonucleic acid
AOPP	Advanced oxidation protein products
GSH	Reduced glutathione
SOD	Superoxide dismutase
CAT	Catalase
GPx	Glutathione peroxidases
GST	Glutathione-S-transferases
GR	Glutathione reductase
LPO	Lipid peroxidation
Cu,Zn-SOD	Copper-Zinc Superoxide Dismutase
Mn-SOD	Manganese Superoxide Dismutase
Trx	Thioredoxin
TrxR	Thioredoxin reductase
NO	Nitric oxide
FAD	Flavin adenine dinucleotide
ETC	Electron transport chain
mPTP	Mitochondrial permeability transition pore
MQC	Mitochondrial quality control
PGC-1α	Peroxisome proliferator-activated receptor gamma coactivator 1-alpha
CRP	C-reactive protein
PAI-1	Plasminogen activator inhibitor-1
BDNF	Brain-derived neurotrophic factor
MDAL	MDA-lysine
SIRT	Sirtuin
mRNA	Messenger Ribonucleic Acid
IDA	Iron deficiency anemia
Mg	Magnesium
PPAR	Peroxisome proliferator-activated receptors (e.g., PPAR-γ)
25(OH)D	25-hydroxycholecalciferol
Se	Selenium
NAC	N-acetylcysteine
