# Correction: Darenskaya et al. Oxidative Stress, Antioxidant Cofactor Micronutrients, and Cognitive Outcomes in Childhood Obesity: Mechanisms, Evidence, and Therapeutic Opportunities. *Int. J. Mol. Sci.* 2025, *26*, 12012

**DOI:** 10.3390/ijms27093921

**Published:** 2026-04-28

**Authors:** Marina Darenskaya, Karen J. Cloete, Luybov Rychkova, Sergey Kolesnikov, Zhanna Prokhorova, Natalya Semenova, Natalya Yuzvak, Lyubov Kolesnikova

**Affiliations:** 1 Scientific Centre for Family Health and Human Reproduction Problems, 664003 Irkutsk, Russiasikolesnikov2012@gmail.com (S.K.); kolesnikova20121@mail.ru (L.K.); 2 UNESCO-UNISA Africa Chair in Nanosciences & Nanotechnology Laboratories, College of Graduate Studies, University of South Africa, Muckleneuk Ridge, P.O. Box 392, Pretoria 0003, South Africa; kaboutercloete@gmail.com

The following reference [93] has been retracted and should be removed from the original publication [1]:

93. Islam, M.R.; Akash, S.; Jony, M.H.; Alam, M.N.; Nowrin, F.T.; Rahman, M.M.; Thiruvengadam, M. Exploring the potential function of trace elements in human health: A therapeutic perspective. *Mol. Cell. Biochem.* **2023**, *478*, s11010–s11022.

With this correction, the order of some references has been adjusted accordingly.

In order to maintain accuracy and precision, a correction has been made to the sentence containing the original reference [93] in Section 5, Microelements as Cofactors of AOD Enzymes, Paragraph Number 1:

Trace elements are essential chemical elements that maintain the homeostasis of the internal environment and perform numerous functions; they are integral components of enzymes, vitamins, hormones, and pigments.

The authors state that the scientific conclusions are unaffected. The corrections were approved by the Academic Editor. The original publication has also been updated.
